# Computational Identification of miRNAs and Temperature-Responsive lncRNAs From Mango (*Mangifera indica* L.)

**DOI:** 10.3389/fgene.2021.607248

**Published:** 2021-06-07

**Authors:** Nann Miky Moh Moh, Peijing Zhang, Yujie Chen, Ming Chen

**Affiliations:** ^1^Biotechnology Research Department, Ministry of Education, Kyaukse, Myanmar; ^2^State Key Laboratory of Plant Physiology and Biochemistry, Department of Bioinformatics, College of Life Sciences, Zhejiang University, Hangzhou, China; ^3^College of Life Sciences and Food, Inner Mongolia University for the Nationalities, Tongliao, China

**Keywords:** mango, *Mangifera indica*, miRNA, lncRNA, stress response, target genes, interaction network

## Abstract

Mango is a major tropical fruit in the world and is known as the king of fruits because of its flavor, aroma, taste, and nutritional values. Although various regulatory roles of microRNAs (miRNAs) and long non-coding RNAs (lncRNAs) have been investigated in many plants, there is yet an absence of such study in mango. This is the first study to provide information on non-coding RNAs (ncRNAs) of mango with the aims of identifying miRNAs and lncRNAs and discovering their potential functions by interaction prediction of the miRNAs, lncRNAs, and their target genes. In this analysis, about a hundred miRNAs and over 7,000 temperature-responsive lncRNAs were identified and the target genes of these ncRNAs were characterized. According to these results, the newly identified mango ncRNAs, like other plant ncRNAs, have a potential role in biological and metabolic pathways including plant growth and developmental process, pathogen defense mechanism, and stress-responsive process. Moreover, mango lncRNAs can target miRNAs to reduce the stability of lncRNAs and can function as molecular decoys or sponges of miRNAs. This paper would provide information about miRNAs and lncRNAs of mango and would help for further investigation of the specific functions of mango ncRNAs through wet lab experiments.

## Introduction

Non-coding RNAs (ncRNAs) are RNA molecules that have no or little protein-coding potential and are not translated into proteins although they are transcribed from DNA. These ncRNAs can be classified according to their length. Small ncRNAs including microRNA (miRNA), small interfering RNA (siRNA), small nucleolar RNA (snoRNA), and piwi-interacting RNA (piRNA) are shorter than 200 nucleotides (nt) in length, and long non-coding RNA (lncRNAs) are longer than 200 nt (Blignaut, 2012).

The miRNAs are small (18–24 nt), endogenous, and regulatory RNA molecules derived from their long self-complementary precursor sequences which can fold into hairpin secondary structures (Ambros et al., 2003). In plants, these long primary precursor miRNAs are transcribed by RNA polymerase II or RNA polymerase III and then processed by dicer-like 1 enzyme (DCL1) into miRNA/miRNA^∗^ duplex which is the mature miRNA sequence and its opposite miRNA strand (miRNA^∗^) (Kurihara and Watanabe, 2004; Panda et al., 2014). Finally, the mature miRNAs are incorporated into an RNA-induced silencing complex (RISC) (Bartel, 2004). The binding of miRNAs to their targeted mRNAs in a perfect or nearly perfect complementarity suggests a method for identifying their targets by BLAST analysis (Zhang et al., 2006a) or other related publicly available tool like psRNATarget^1^ (Dai and Zhao, 2011). Many experimental researches have proved that miRNAs are involved in many important biological and metabolic processes. In plants, miRNAs play a fundamental role in almost all biological and metabolic processes including plant growth, development, signal transduction, and various stress responses by binding to their target genes (Rhoades et al., 2002).

Long non-coding RNAs are a family of regulatory RNAs having a minimal length of 200 nt. Most lncRNAs are transcribed by RNA polymerase II although some are transcribed by RNA polymerase III (Dieci et al., 2007; Geisler and Coller, 2013; Zhang and Chen, 2013). LncRNAs can interact with ncRNAs such as miRNAs (Jalali et al., 2013). LncRNAs not only can target miRNAs to reduce the stability of lncRNAs but also can function as molecular decoys or sponges of miRNAs (Salmena et al., 2011). Moreover, lncRNAs can compete with miRNAs to bind to their target mRNAs and are the precursors for the generation of miRNAs to silence target mRNAs (Yoon et al., 2014). Many lines of evidence showed that plant lncRNAs play an important role in fundamental biological processes including growth and development and abiotic stress responses (Xin et al., 2011). However, the molecular basis of how lncRNAs function and mediate gene regulation is still poorly understood (Megha et al., 2015).

The genus *Mangifera* belongs to the family Anacardiaceae and contains about 69 different species. *Mangifera indica* L. (mango) is the most common species among them (Mukherjee, 1972; Slippers et al., 2005). Mango is one of the main tropical fruits over the world and is believed to have originated from Asia (Hirano et al., 2010). The well-known countries for mango cultivation are China, India, Thailand, Pakistan, Mexico, Philippines, and Myanmar. The annual production of mango is approximately 42 million tons which is second after banana production (Galán Saúco, 2010). Mango is called as the king of fruits because of its special characteristic flavor, pleasant aroma, taste, and nutritional values. Both ripe and raw fruits can be used as food products such as pickles, juice, jam, powder, sauce, cereal flakes, and so on (Siddiq et al., 2012). Moreover, various parts of mango trees have been used for medical purposes a long time ago, mostly in Southeast Asian and African countries (Mukherjee, 1953). *In vitro* and *in vivo* studies have indicated the various pharmacological potentials of *M. indica* such as anticancer, anti-inflammatory, antidiabetic, antioxidant, antifungal, antibacterial, anthelmintic, gastroprotective, hepatoprotective, immunomodulatory, antiplasmodial, and antihyperlipidemic effects (Lauricella et al., 2017). As a tropical plant, mango is susceptible to cold temperature (Sudheeran et al., 2018), and its floral morphogenesis, photosynthesis, and stomatal limitation are induced by chilling temperature (Núñez-Elisea and Davenport, 1994; Allen et al., 2000). Heat treatment can also affect mango genes involved in stress response and pathogen defense mechanism, genes involved in chlorophyll degradation and photosynthesis, and genes involved in sugar and flavonoid metabolism (Luria et al., 2014).

Although mango is a popular plant with many important usages, its ncRNA data are still limited. Over 10,000 miRNA data of several plants can be accessed in the miRNA database, miRBase^2^, but mango miRNAs and their functions have not yet been identified. The regulatory roles of lncRNAs and the molecular basis of lncRNA-mediated gene regulation are also still poorly understood in plants including mango. So, the aims of this research work are to identify and study about the miRNAs and lncRNAs of mango and to examine their potential functions by the interaction prediction of the miRNAs, lncRNAs, and their target genes.

## Materials and Methods

### Data Collection

A total of 10,415 plant miRNAs (release 21) were downloaded from the miRBase database (see text footnote 2), and the redundancy sequences were removed. The resulting 6,042 non-redundant known miRNAs were used as the reference for the prediction of conserved miRNAs.

For the identification of mango miRNAs, 107,744 mango unigenes collected from the mango RNA-Seq database^3^ were used (Tafolla-Arellano et al., 2017). These unigenes were derived from the peels of Keitt mango cultivar.

A total of 277,071 RNA transcripts from Zill (Wu et al., 2014), Shelly (Luria et al., 2014; Sivankalyani et al., 2016), and Keitt (Tafolla-Arellano et al., 2017) mango cultivars were used for the identification of lncRNAs.

### Identification of miRNAs and Their Precursors

**FIGURE 1:**
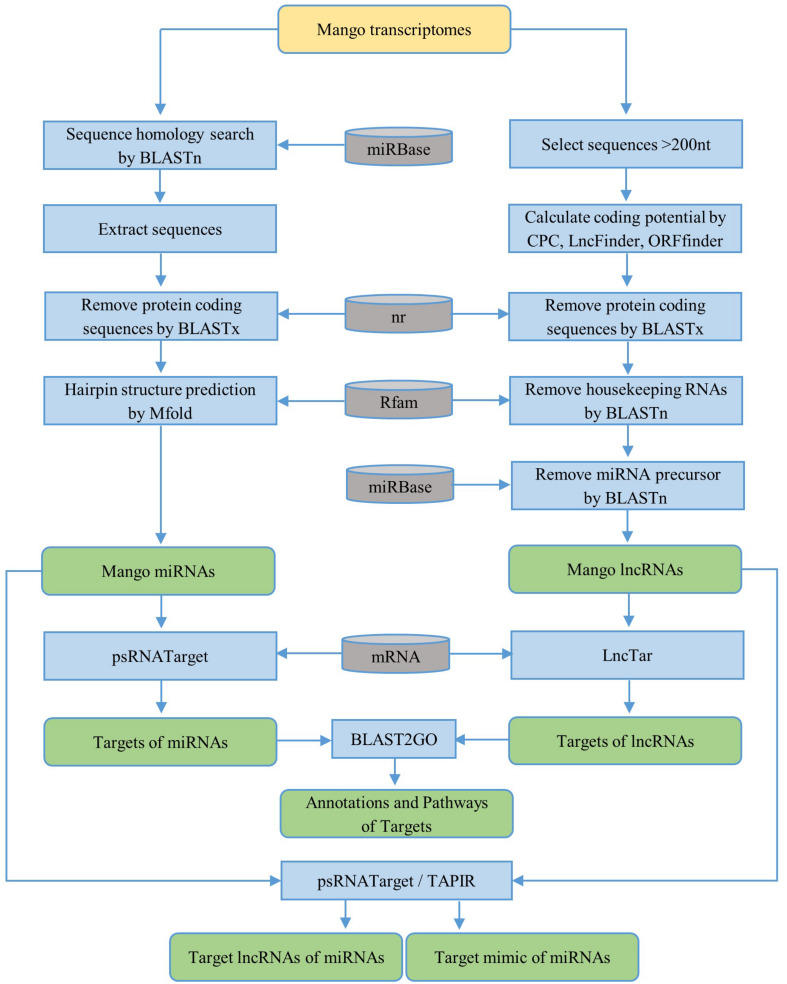
Workflow for the identification of ncRNAs [microRNAs (miRNAs) and long non-coding RNAs (lncRNAs)] and their targets. The yellow rounded rectangle represents the data input and the green rounded rectangles represent the data output. The blue rectangles are the processing steps and the gray cans represent databases.

First, the homology search of mango unigenes against non-redundant plant miRNAs was performed by using BLASTn (BLAST+ 2.7.1) with an *e*-value cutoff of 10. The following criteria were used to choose the candidate miRNAs: the length of the candidate miRNA should be greater than or equal to 18 nt without gap and the number of mismatches between mango sequences and plant miRNAs should not be more than 2. The sequences of 100 nt upstream and 100 nt downstream from the BLAST hit were extracted for precursor sequences. If the length of the query sequence was less than 200 nt, the entire sequence was selected. BLASTx against NCBI non-redundant (nr) protein databases was used to remove the protein-coding sequences from the extracted precursor sequences with an *e*-value cutoff of 0.01. The secondary structures of the remaining precursor sequences were predicted by using the Zuker folding algorithm in MFOLD (version 2.3) software (Zuker, 2003) with default parameters. The workflow for the identification of miRNAs is briefly described in Figure 1. Based on the parameters suggested by Dr. Zhang in the identification and characterization of new plant miRNAs using EST analysis (Zhang et al., 2005), the potential pre-miRNAs were predicted as follows:

1. The minimum length of precursor should be at least 60 nt;
2. The pre-miRNA sequence should be folded into an appropriate stem-loop hairpin secondary structure;
3. It should contain the mature miRNA within one arm of the hairpin;
4. The predicted mature miRNAs and its opposite miRNA^∗^ sequence in the other arm of the hairpin should not have more than 6 nt mismatches;
5. Loop or break should not be contained between the miRNA/miRNA^∗^ duplex;
6. Maximum size of a bulge in the mature miRNA sequences should not be more than 3 nt;
7. The predicted secondary structures should have higher minimal free energies (MFE) and minimal free energy index (MFEI);
8. MFEI of pre-miRNA should be greater than 0.7;
9. A+U content should be within 30–70%.

The following equations were used to calculate the MFEI and adjusted minimal free energy (AMFE):

$$\mathrm{AMFE}=\left(\frac{\text{MFE}}{\text{length}\,\text{of}\,\text{pre}-\text{miRNA}}\right)\times 100$$

$$\mathrm{MFEI}=\frac{\text{AMFE}}{\left(G+C\right)\%}$$

### Prediction of Candidate lncRNAs

To predict the lncRNAs, the transcripts smaller than 200 nt were firstly removed. The coding potential of the remaining transcripts was then calculated by CPC (coding potential calculator) (Kong et al., 2007) and LncFinder (Han et al., 2019). Only sequences with a CPC score less than -1 and LncFinder score less than 0.5 were used for further prediction. The protein coding sequences were removed by BLASTx against the NCBI nr protein databases. Also, the ORF Finder tool^4^ was used to predict the open reading frame (ORF) of the remaining sequences, and the minimal ORF cutoff less than 102 amino acids was applied for the prediction. Then, housekeeping genes were removed against the Rfam_14.0 database^5^ with *e*-value 0.001. Finally, to remove the lncRNAs acting as precursors of known or novel miRNAs, lncRNAs were aligned with precursors of known non-redundant plant miRNAs from the miRBase database^6^ using BLASTn with default parameters (Figure 1).

The remaining transcriptome sequences that were not captured as lncRNAs were used as queries against the NCBI nr protein database using BLASTx with a cutoff *e*-value of 1e-5. The sequences with BLAST hits were then analyzed to remove the housekeeping RNAs. The final sequences were identified as protein coding sequences in this study for target gene analysis.

### Identification of Significantly Expressed Temperature-Responsive lncRNAs

Mango is a tropical plant and is susceptible to cold temperature (Sudheeran et al., 2018), and its floral morphogenesis, photosynthesis, and stomatal limitation are induced by chilling temperature (Núñez-Elisea and Davenport, 1994; Allen et al., 2000). A high temperature can also affect mango genes involved in stress response and pathogen defense mechanism, genes involved in chlorophyll degradation and photosynthesis, and genes involved in sugar and flavonoid metabolism (Luria et al., 2014). Therefore, temperature-responsive lncRNAs were identified. From the resulting lncRNA transcripts, the temperature-responsive lncRNAs were filtered by two parameters. The mango lncRNAs with an adjusted *p*-value of 0.05 and a log2 fold change of greater than 2 or less than -2 were identified as the significantly expressed lncRNAs.

### Target Gene Prediction of miRNAs and lncRNAs

Mango mRNAs downloaded from the NCBI database and mango protein coding sequences previously identified were used for the target gene prediction of miRNAs. The putative target sites of miRNAs were identified by aligning miRNA sequences using the plant target prediction tool, psRNATarget (2017 release) server (see text footnote 1) (Dai and Zhao, 2011). To reduce the number of false predictions, the maximum expectation threshold was set to the value of 3.0. The cutoff length of nucleotides for complementarity scoring, hsp (high-scoring segment pair) size, was set as the length of the mature miRNAs. The maximum energy of unpairing (UPE) of the target site was set as 25 kcal. The flanking length around the target site for target accessibility analysis was 17 bp upstream and 13 bp downstream. The range of central mismatch leading to translation inhibition was adjusted as 9–11 nt. No gap and no more than four mismatches between miRNA and its target (G-U pair count as 0.5 mismatch) were allowed. The target genes of mango lncRNAs were predicted by using the LncTar tool (version 1.0) (Li et al., 2015) with the normalized binding free energy (ndG) cutoff value less than 0.1.

### Prediction of lncRNAs as miRNA Target or Target Mimic

To predict the lncRNAs as the target genes of miRNAs, psRNATarget (2017 release) (Dai and Zhao, 2011) was used as previously mentioned in the interaction prediction of miRNAs and mRNAs. For target mimic prediction, the TAPIR server (version 1.2) (Bonnet et al., 2010) was used in this study. TAPIR is a web server for the prediction of plant miRNA targets including target mimics.

### Functional Annotation and Pathway Analysis of Target Genes

The gene ontology (GO) analysis of the identified target transcripts was executed by combining both BLASTx data and InterProScan analysis data by means of the BLAST2GO_5.2.5 software (Conesa et al., 2005). The GO enrichment analysis was done by using Fisher’s exact test with multiple testing correction of false discovery rate (FDR). The Kyoto Encyclopedia of Genes and Genomes (KEGG) pathway analysis was also performed for a better understanding of the functions of the target genes.

### Conservation Analysis of lncRNAs

The analysis of the conservation of mango lncRNAs was detected by using BLASTn against all lncRNA sequences from the plant lncRNA database, CANTATAdb_2.0 (Szcześniak et al., 2016), with *e*-value cutoff 1e-20.

### Interaction Network of miRNAs, lncRNAs, and Their Target Genes

Finally, the interaction network of miRNAs, lncRNAs, and their target genes was visualized by using Cytoscape_3.7.2 (Shannon et al., 2003).

## Results

### Identification and Characterization of Mango miRNAs

Most of the plant miRNAs are evolutionarily conserved from species to species (Dezulian et al., 2005; Weber, 2005), and this indicates the powerful strategy for the identification of new miRNAs by using the already known miRNAs (Zhang et al., 2006b). Many conserved miRNAs have been identified from the expressed sequence tag (EST) (Zhang et al., 2005; Frazier and Zhang, 2011) and genome survey sequence (GSS) (Pan et al., 2007) by using this homology search approach. For mango, there are no GSS data and the available EST data for mango were only 1,709 and it was not sufficient for the identification of miRNA. Hence, unigenes (107,744) (see text footnote 3) were used for the identification of miRNAs in this study. Unigene is a unique transcript that is transcribed from a genome, and many miRNAs have been identified from the unigenes of many plant species such as *Artemisia annua* (Pérez-Quintero et al., 2012), coconut (Naganeeswaran et al., 2015), litchi fruit (Yao et al., 2015), and black pepper (Asha et al., 2016).

**FIGURE 2:**
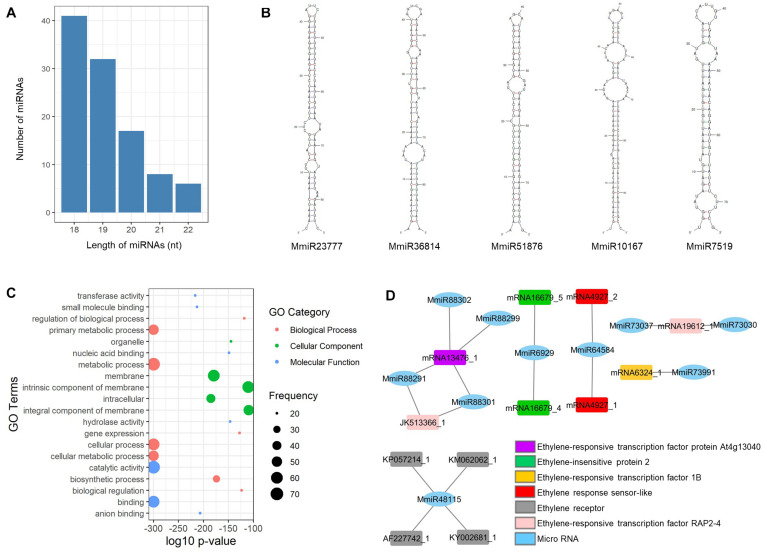
Mango miRNAs. **(A)** The number and length distribution of miRNAs; **(B)** hairpin structures of five precursor miRNAs involved in the developmental process of mango; **(C)** GO enrichment analysis of highly enriched top 20 target genes of miRNAs; **(D)** interaction among the 10 mango miRNAs and six ethylene-responsive target genes.

From the mango unigenes, we have identified 104 miRNAs by following the identification workflow explained in Figure 1. The length of the resulting mature miRNAs is in the range of 18–22 nt. Among them, nearly 40% (41 miRNAs) of mango mature miRNAs are in the length of 18 nt and 6 miRNAs have the length of 22 nt. Thirty-two miRNAs, 17 miRNAs, and 8 miRNAs are 19, 20, and 21 nt of length, respectively (Figure 2A). The potential 104 pre-miRNAs of mango were predicted based on the parameters by Dr. Zhang (Zhang et al., 2005), and the MFEI values were also calculated as the MFEI gave the best prediction of miRNAs (Zhang et al., 2006c). The precursor length of mango miRNAs (MmiRs) was varied significantly from 67 to 144 nt with an average length of 94 nt. We denoted the name of mango miRNA as MmiR with the numbers. The secondary structure of precursor sequences was predicted by Zuker folding algorithm in MFOLD. Figure 2B shows the hairpin structures of five miRNAs (MmiR23777, MmiR36814, MmiR51876, MmiR10167, and MmiR7519) involved in the developmental process of mango according to the result of GO enrichment and KEGG pathway analysis. The average MFE of pre-miRNAs is 29.92. The MFEI values were also calculated and were in the range of 0.7–1.45 with the average MFEI of 0.84. The A+U content was in the range of 42–70% with an average of 62.9% (Supplementary Table 1).

### Target Gene Analysis of miRNAs

According to the result of target gene prediction by the psRNATarget server (Dai and Zhao, 2011), all the newly identified mango miRNAs could bind to their targets and a total of 2,347 target genes were predicted for 104 mango miRNAs. The predicted target genes were annotated and assigned to GO terms by BLAST2GO. The top 20 highly enriched GO terms of miRNA target genes are visualized in Figure 2C. The metabolic process and cellular process are highly enriched GO terms of the biological process, and membrane and intracellular are highly enriched GO terms of the cellular component. In molecular function, catalytic activity and binding are highly enriched GO terms.

From KEGG pathway analysis, 310 targets were involved in the 103 different KEGG pathways. Purine metabolism was the pathway with the highest target genes: 136. The predicted miRNAs, their target genes, target descriptions, target GO terms, and target KEGG pathways are shown in Supplementary Table 2.

### Identification and Characterization of Mango lncRNAs

For the identification of lncRNAs, a total of 277,071 RNA transcripts from Zill (Wu et al., 2014), Shelly (Luria et al., 2014; Sivankalyani et al., 2016), and Keitt (Tafolla-Arellano et al., 2017) mango cultivars were used. First, the sequences less than 200 nt were removed because lncRNAs were always longer than 200 nt. Then, the coding transcripts were removed by their protein-coding potential, homology with known proteins, and potential ORFs. Finally, the housekeeping RNAs and the precursor of miRNAs were removed. After a series of filtering steps, a total of 31,226 candidate lncRNAs were predicted.

**FIGURE 3:**
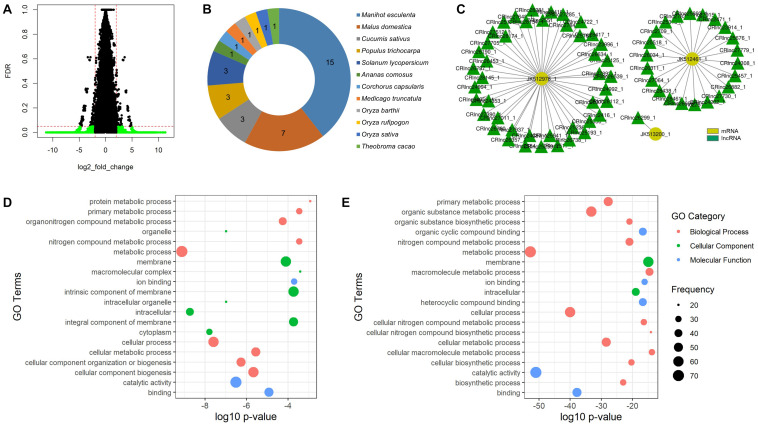
Temperature-responsive lncRNAs of mango. **(A)** Volcano plot of cold-responsive lncRNAs of mango; green plots represent significantly expressed cold-responsive lncRNAs with false discovery rate (adjusted *p*-value) of less than 0.05 and log2 fold change of less than –2 or greater than 2; **(B)** conserved number of mango lncRNAs in different plant species; **(C)** interaction subnetwork between three low temperature-responsive proteins and cold-responsive lncRNAs of mango; **(D)** GO enrichment analysis of highly enriched genes targeted by heat-responsive lncRNAs; **(E)** GO enrichment analysis of highly enriched target genes of cold-responsive lncRNAs.

The temperature-responsive lncRNAs were then defined by fold change value and FDR adjusted *p*-value to filter out the significantly expressed mango lncRNAs. The lncRNAs with fold change value less than -2 were defined as the downregulated lncRNAs and the lncRNAs greater than 2 as the upregulated lncRNAs. The FDR adjusted *p*-value was set to 0.05. As a result, 24 lncRNAs were significantly expressed to heat stress (55°C hot water brushing) and 7,586 lncRNAs to cold stress (5, 8, or 12°C) (Figure 3A). We denoted the name of the heat-responsive lncRNAs of mango as HRlnc with numbers and the cold-responsive lncRNAs as CRlnc with numbers. 24 HRlncRNA sequences are shown in the Supplementary Data Sheet 1 and 7,586 CRlncRNA sequences are in the Supplementary Data Sheet 2. In heat-responsive lncRNAs, 18 lncRNAs were upregulated and 6 lncRNAs were downregulated. The length of heat-responsive lncRNAs ranged from 213 to 1,186 nt (Supplementary Table 3). Among the 7,619 cold-responsive lncRNAs, 4,335 were upregulated and 3,251 were downregulated. The length of cold-responsive lncRNAs was in the range of 201–2,746 nt (Supplementary Table 4).

### Conservation Analysis of lncRNAs

The mango lncRNAs were searched by using BLASTn against the plant lncRNA database, CANTATAdb, with *e*-value cutoff 1e-20 to check their evolutionary conservation. As a result, no heat-responsive lncRNAs were conserved and 22 cold-responsive lncRNAs were conserved with 12 different plant species. Among these different plants, *Manihot esculenta* (cassava) is the most highly conserved plant and 15 cold-responsive mango lncRNAs were conserved with its lncRNAs (Figure 3B).

### Target Gene Prediction of lncRNAs

To analyze the interaction of the newly identified lncRNAs of mango with protein-coding genes, the lncRNA target prediction tool LncTar was used. A total of 1,998 mango mRNAs downloaded from NCBI were used for the target prediction of lncRNAs. From the resulting data, 6,975 lncRNAs interacted with 1,985 target mRNAs. To analyze the functional overview of the identified lncRNAs, the targets of the identified lncRNAs were predicted by BLAST2GO. Among the 24 heat-responsive lncRNAs, 8 lncRNAs had 115 target genes (SCL14, STP13, Hsp70, At4g39970, ACO1, and so on) involved in plant development and stress response. In cold-responsive lncRNAs, 6,951 lncRNAs interacted with 1,985 target genes. The WRKY proteins are a large family of transcriptional regulators in higher plant and 64 cold-responsive lncRNAs interact with the WRKY gene family in this study (Figure 3C).

Moreover, functional prediction of the target genes of the identified lncRNAs was performed by GO enrichment analysis. For the target genes of heat-responsive lncRNAs, metabolic process, cellular process, cellular component biogenesis, and cellular metabolic process were highly enriched in the biological process. In the cellular component analysis, GO terms associated with membranes and intracellular were highly enriched. Catalytic activity and binding GO terms were highly enriched in molecular function analysis (Figure 3D). For the target genes of cold-responsive lncRNAs, metabolic processes and cellular processes were highly enriched for biological processes. In the cellular component analysis, GO terms related to membranes and intracellular were highly enriched. For molecular function analysis, most of the enriched GO terms were related to catalytic activity and binding (Figure 3E).

From the results of the KEGG pathway analysis, heat-responsive lncRNAs had target genes involved in 17 KEGG pathways (Supplementary Table 5). Among these different pathways, amino sugar and nucleotide sugar metabolism was the most significant pathway and eight target genes were involved in this pathway. For cold-responsive lncRNAs, 209 target genes had been mapped to 87 KEGG pathways (Supplementary Table 6). JK513026_1, alcohol dehydrogenase 1 (ADH1, EC:1.1.1.1), was the most enriched target gene and involved in 12 different pathways.

### Prediction of lncRNAs as miRNA Targets

To analyze the direct interaction of miRNAs and lncRNAs of mango, the psRNATarget server (Dai and Zhao, 2011) was used to predict the target lncRNAs of miRNAs. The resulting data showed that three heat-responsive lncRNAs interacted with six miRNAs (Supplementary Table 7). For cold-responsive lncRNAs, 763 lncRNAs had 1,203 pairs of interactions with 89 miRNAs (Supplementary Table 8).

**FIGURE 4:**
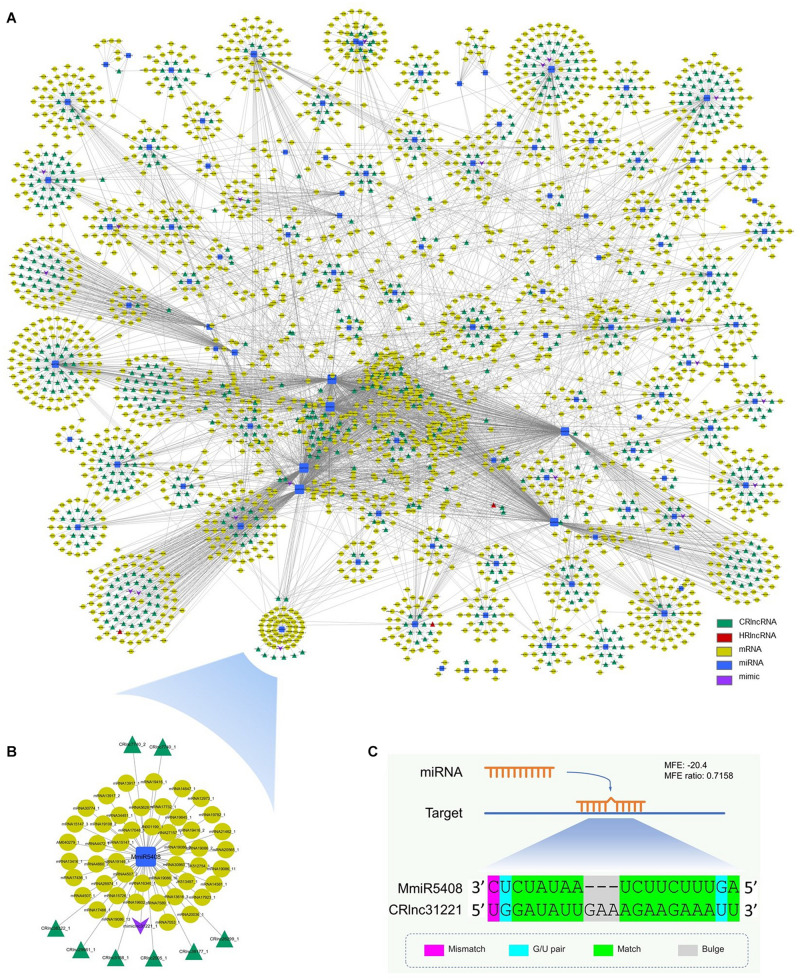
Interaction network among mango ncRNAs and their targets; green triangles represent cold-responsive lncRNAs, red triangles represent heat-responsive lncRNAs, yellow ellipses are used for target genes, blue rectangles are for miRNAs, and purple V-shapes are for target mimics. **(A)** Network of interaction among newly identified miRNAs, newly identified lncRNAs (cold-responsive lncRNAs and heat-responsive lncRNAs), and newly identified target mimic of miRNAs and mRNAs; **(B)** subnetwork of interaction among MmiR5408, its target mimic CRlnc31221, target CRlncRNAs, and 47 target genes; **(C)** schematic diagram of the interaction between MmiR5408 and its target mimic cold-responsive lncRNA, CRlnc31221.

The miRNA target mimicry search was also performed by using TAPIR. No heat-responsive lncRNA acts as the target mimic of miRNAs. However, 20 cold-responsive lncRNAs were predicted as the target mimics of 20 miRNAs (Supplementary Table 9). CRlnc31221 was the target mimic of MmiR5408 which targeted eight cold-responsive lncRNAs and 47 target genes (Figure 4B). The schematic diagram of the interaction between MmiR5408 and its target mimic cold-responsive lncRNA, CRlnc31221, is shown in Figure 4C.

The interaction network of mango ncRNAs (miRNAs, lncRNAs, and mimic) and their target genes was visualized by using Cytoscape, which contained a total of 5,388 pairs of interaction among miRNAs, lncRNAs, and their targets (Figure 4A). These interactions were 4,155 pairs of 104 miRNAs and 2,347 mRNAs, 1,203 pairs of 89 miRNAs and 763 cold-responsive lncRNAs, six pairs of six miRNAs and four heat-responsive lncRNAs, and 24 pairs of 20 miRNAs and their 20 target mimics.

## Discussion

### Identification, Characterization, and Target Gene Prediction of miRNAs

The length of the predicted 104 mature miRNAs was in the range of 18–22 nt, but the length of precursor miRNAs varied significantly from 67 to 144 nt with an average length of 94 nt. The predicted mango miRNAs belong to 86 different families. Among them, over 70% of the miRNA families have only one family member. The highest five family members were found in the miR2673 family followed by miR159, with four family members. The remaining miRNAs have a family member of two or three. Therefore, we can see that the mango miRNA distribution across various families is highly heterogeneous.

Previous studies have already proved that plant miRNAs bind to their targets in a perfect or nearly perfect complementarity (Bartel, 2004, Zhang et al., 2006a) and the psRNATarget server (Dai and Zhao, 2011) was used to search the target gene of mango miRNAs in this study. Both mRNAs collected from NCBI and identified in this study were used as the target candidates of miRNAs due to the absence of *M. indica* target candidates in the psRNATarget server. Some previous studies indicated that miR156 was a master regulator of the juvenile phase in plants and it targeted the Squamosa promoter binding protein-Like (SPL) gene family to regulate the transition from vegetative phase to floral phase in *Arabidopsis* (Gandikota et al., 2007; Wu et al., 2009; Yamaguchi et al., 2009), maize (Chuck et al., 2007) and rice (Jiao et al., 2010; Jeong et al., 2011). In mango, MmiR105772, a family of miR156, also bound to its target SPL6, and thus, the predicted targets of mango miRNAs were in the agreement with the previously published papers in other plants. The resulting data from psRNATarget also showed that only one miRNA (MmiR1653) had a single target gene which was a member of the miR482 family and bound to monodehydroascorbate reductase four enzyme, an important gene related to the nutritional quality of mango fruit (Pandit et al., 2010). All the other miRNAs could target multiple genes and some miRNAs had over 200 target genes. For example, MmiR73030 had 230 target genes and these target genes are involved in 16 KEGG pathways such as biosynthesis of antibiotics, purine metabolism, sulfur metabolism, glycerophospholipid metabolism, T cell receptor signaling pathway, steroid degradation, and so on.

Sivankalyani et al. (2016) published that the mango stress-response pathways were activated by cyclic nucleotide-gated channel (CNGC) and leucine-rich repeat receptor (Lrr). In this study, we found that MmiR90392 targeted CNGC1, and MmiR68471 and MmiR68478 targeted Lrr2. Moreover, MmiR10167 and MmiR15558 are bound to the stress WRKY transcription factor 44 which plays a major role in plant defense to biotic and abiotic stresses. MmiR78769 and MmiR101928 are also bound to their target genes of phospholipase A and phospholipase D which were key factors in plant responses to biotic and abiotic stresses (Xue et al., 2007). The ethylene response could improve the tolerance of mango fruit to chilling stress (Lederman et al., 1997), and 10 mango miRNAs identified in this study had six ethylene-responsive target genes such as ethylene-insensitive protein and ethylene-responsive transcription factor (Figure 2D). So, these newly identified mango miRNAs have potential roles in the chilling stress-responsive process of mango.

Two mango miRNAs (MmiR23777 and MmiR36814) also targeted the auxin efflux carrier which had a potential role in mango plant organ development (Li et al., 2012). A total of 17 miRNAs interacted with auxin-related genes. MmiR51876 was a miRNA that targeted auxin-responsive protein. The pentose and glucuronate interconversion pathway, phenlypropanoid biosynthesis pathway, and alpha-linolenic acid metabolism pathway were KEGG pathways involved in the adventitious root formation of mango cotyledon segments (Li et al., 2017). In this study, nine miRNAs bound to eight target genes are involved in these three pathways for mango root formation. MmiR10167 bound to target genes is involved in phenlypropanoid biosynthesis pathway and MmiR7519 bound to target genes is involved in alpha-linolenic acid metabolism pathway. From these findings, it was observed that these five mango miRNAs (MmiR23777, MmiR36814, MmiR51876, MmiR10167, and MmiR7519) are involved in the developmental process of mango (Figure 2B).

### Identification, Characterization, and Target Gene Prediction of lncRNAs

As the genome sequence of mango is not available till now, the *de novo* assembled transcriptome sequences were used for the identification of lncRNAs in this study. A total of 277,071 RNA transcripts from Zill (Wu et al., 2014), Shelly (Luria et al., 2014; Sivankalyani et al., 2016), and Keitt (Tafolla-Arellano et al., 2017) mango cultivars studied by former researchers were used, and a total of 31,226 candidate lncRNAs were predicted in this study. Heat and cold stresses can affect the important mechanisms of mango such as stress response, defense mechanism, sugar and flavonoid metabolism, photosynthesis, and floral morphogenesis (Núñez-Elisea and Davenport, 1994; Allen et al., 2000; Luria et al., 2014; Sudheeran et al., 2018). Therefore, the temperature-responsive lncRNAs were identified from the resulting 31,226 lncRNAs. Among them, 24 lncRNAs were significantly expressed to heat stress and 7,586 lncRNAs to cold stress. The most significantly expressed downregulated heat-responsive lncRNA was HRlnc25944 with a fold change value of 6.22. HRlnc11351 and HRlnc27371 were the mostly expressed upregulated lncRNAs with a fold change value greater than 7. For the cold-responsive lncRNAs, CRlnc10871 was the mostly expressed downregulated lncRNA (FC value -11.19), and CRlnc26299, CRlnc30496, and CRlnc36473 were the most significantly expressed lncRNAs with a fold change value greater than 11.

No heat-responsive lncRNAs were conserved, but 0.29% of cold-responsive lncRNAs were conserved with 12 different plant species (Figure 3B). Among them, the highest conserved lncRNAs were CRlnc32663 and CRlnc47883, each of which was conserved with four different lncRNAs of other plants. CRlnc32663 was conserved with four different lncRNAs of three different plant species such as *Manihot esculenta* (cassava), *Malus domestica* (apple), and *Populus trichocarpa* (the black cottonwood). CRlnc47883 was also conserved with four lncRNAs of *Oryza rufipogon* (brownbeard rice), *Oryza barthii* (wild rice), and *Solanum lycopersicum* (tomato).

For heat-responsive lncRNAs, 8 bound to 115 target genes were involved in plant development and stress response. HRlnc11351 was the most significantly expressed upregulated lncRNAs with a fold change value of 7.55 and bound to six heat shock proteins. In cold-responsive lncRNAs, CRlnc26299 was one of the most significantly expressed upregulated lncRNAs and bound to RC12B (JK513200_1), which is a low-temperature and salt-responsive protein found in *Arabidopsis thaliana* (Medina et al., 2001). The WRKY proteins are a large family of transcriptional regulators in higher plants and exhibited variable expression patterns in response to chilling stress in cucumber (Ling et al., 2011), mango (Sivankalyani et al., 2016), and rice (Ramamoorthy et al., 2008). In this study, 64 cold-responsive lncRNAs interact with the WRKY gene family. So, we can observe that the cold-responsive lncRNAs of mango have interaction with the target genes that are expressed at low-temperature stress.

Gene ontology enrichment analysis and KEGG pathway analysis were performed for a better understanding of the target genes of newly identified lncRNAs. From the GO enrichment analysis result, we could see that both types of heat-responsive lncRNAs and cold-responsive lncRNAs were highly enriched in metabolic processes and cellular processes in the biological process analysis. In the cellular component analysis, GOs related to membranes, intracellular, and cytoplasm were highly enriched for both types of lncRNAs. Meanwhile, most of the enriched GO terms in both types of lncRNAs were related to catalytic activity and binding for molecular function analysis. Therefore, we could see that the GO terms highly enriched in both heat-responsive and cold-responsive lncRNAs were not quite different.

Among the 17 KEGG pathways of the target genes of the heat-responsive lncRNAs, amino sugar and nucleotide sugar metabolism was the most significant pathway, and eight target genes were involved in this pathway. As mentioned above, HRlnc11351 was the most significantly expressed upregulated lncRNAs and its target gene, JK513625_1, is 3-ketoacyl-CoA thiolase 2 (KAT2, EC:2.3.1.16), which could be mapped to nine different pathways such as benzoate degradation; fatty acid elongation; biosynthesis of unsaturated fatty acids; alpha-linolenic acid metabolism; fatty acid degradation; valine, leucine, and isoleucine degradation; biosynthesis of antibiotics: geraniol degradation; and ethylbenzene degradation according to the result of the KEGG pathway analysis. In *Arabidopsis*, KAT2 is an enzyme that catalyzes the β-oxidation of fatty acid and involves in abscisic acid (ABA) signal transduction (Jiang et al., 2011). The phytohormone ABA plays an important role in plant development and adaptation to diverse environmental stresses. Therefore, HRlnc11351 may be involved and played an important role in mango development and stress response by targeting KAT2. For cold-responsive lncRNAs, 209 target genes had been mapped to 86 KEGG pathways. JK513026_1, alcohol dehydrogenase 1 (ADH1, EC:1.1.1.1), was the most enriched target gene and involved in 12 different pathways including glycolysis/gluconeogenesis; metabolism of xenobiotics by cytochrome P450; glycine, serine, and threonine metabolism; methane metabolism; fatty acid degradation; and so on. In plants, ADH genes are involved in mediating stress responses and developments. In mango, ADH1 has an important role in fruit ripening (Singh et al., 2010), and thus, cold-responsive lncRNAs that target the ADH1 gene may play an important role in the mango fruit ripening process. According to the KEGG pathway analysis results, purine metabolism and biosynthesis of antibiotics were the highly enriched pathways among 86 pathways and more than 50 target genes were enriched in each pathway.

### Interaction Between lncRNAs and miRNAs

The interaction between miRNAs and lncRNAs showed that most of the miRNAs had targeted more than one lncRNAs and only eight miRNAs had single target lncRNAs. The number of lncRNAs targeted by a single miRNA was in the range of 1–90. A total of 90 target lncRNAs were found for MmiR73030, which also targeted 230 mRNAs. This miRNA had the highest target numbers in both lncRNAs and mRNAs.

Long non-coding RNAs not only can be targeted by miRNAs to reduce the stability of lncRNAs but also can function as molecular decoys or sponges of miRNAs (Salmena et al., 2011; Wu et al., 2013). So, the miRNA target mimicry search was performed by using TAPIR, which is a web server for the prediction of plant miRNA targets including target mimics. Although no heat-responsive lncRNA acts as the target mimic of miRNAs, 20 cold-responsive lncRNAs were predicted as the target mimics of 20 miRNAs. CRlnc31221 was the target mimic of MmiR5408 which targeted 8 cold-responsive lncRNAs and 47 target genes. These target genes were involved in starch and sucrose metabolism, inositol phosphate metabolism, and phenylpropanoid biosynthesis pathways, which are important for plant growth and development and the plant’s response toward biotic and abiotic stresses. During target mimicry, the interactions between miRNAs and their authentic targets were blocked by binding of decoy RNA to miRNAs *via* partially complementary sequences (Franco-Zorrilla et al., 2007). So, the target mimicry of CRlnc31221 had the potential regulation effect to the interaction between the target genes and MmiR5408 (Figure 4B).

## Conclusion

In conclusion, this study identified 104 miRNAs and 7,610 temperature-responsive lncRNAs from mango transcriptome sequences, and the interactions of these ncRNAs with their target genes were also predicted. MmiR105772 is bound to SPL6 gene that regulates the transition from the vegetative phase to the floral phase of plants. MmiR1653 is bound to monodehydroascorbate reductase 4 enzyme that regulates the nutritional quality of mango fruit. MmiR78769 and MmiR101928 are also bound to their target genes of phospholipase A and phospholipase D which were key factors in plant responses to biotic and abiotic stresses. HRlnc11351 may be involved and has an important role in mango development and stress response by targeting KAT2, an enzyme that catalyzes β-oxidation of fatty acid and is involved in abscisic acid (ABA) signal transduction. Cold-responsive lncRNAs that target the ADH1 gene may play an important role in mango fruit ripening process because ADH1 has an important role in mango fruit ripening. CRlnc26299, one of the most significantly expressed upregulated cold-responsive lncRNAs, is bound to RC12B (JK513200_1), which is the low-temperature and salt-responsive protein. According to these results, the newly identified mango ncRNAs, like other plant ncRNAs, have a potential role in metabolic pathways including plant growth and developmental process, pathogen defense mechanism, and stress-responsive process. Therefore, the resulting data of this project may help for further prediction of the specific functions of mango ncRNAs through wet lab experiments.

## Data Availability Statement

The original contributions presented in the study are included in the article/Supplementary Material, further inquiries can be directed to the corresponding author/s.

## Author Contributions

NM and MC designed the research. NM and PZ performed the bioinformatics analysis. NM and YC analyzed the plant gene data. All authors approved the final manuscript.

## Conflict of Interest

The authors declare that the research was conducted in the absence of any commercial or financial relationships that could be construed as a potential conflict of interest. The reviewer, YO declared a past co-authorship with one of the authors, MC, to the handling editor.

## Footnotes

1 http://plantgrn.noble.org/psRNATarget/

2 http://www.mirbase.org/cgi-bin/browse.pl

3 http://bioinfo.bti.cornell.edu/cgi-bin/mango/index.cgi

4 https://www.ncbi.nlm.nih.gov/orffinder/

5 http://rfam.xfam.org

6 http://www.mirbase.org/

## Supplementary Material

Supplementary Table 1 miRNAs data.

Supplementary Table 2 miRNAs targets data.

Supplementary Table 3 Heat responsive lncRNAs data.

Supplementary Table 4 Cold responsive lncRNAs data.

Supplementary Table 5 KEGG pathways of the target of heat responsive lncRNAs.

Supplementary Table 6 KEGG pathways of the target of cold responsive lncRNAs.

Supplementary Table 7 Heat responsive lncRNAs as target of miRNAs.

Supplementary Table 8 Cold responsive lncRNAs as target of miRNAs.

Supplementary Table 9 Cold responsive lncRNAs as target mimic of miRNAs.

Supplementary Data Sheet 1 Heat responsive lncRNAs sequences.

Supplementary Data Sheet 2 Cold responsive lncRNAs sequences.

## Abbreviations

ABA: abscisic acid
ACO1: 1-aminocyclopropane-1-carboxylate oxidase 1
ADH1: alcohol dehydrogenase 1
AMFE: adjusted minimal free energy
BLAST: basic local alignment search tool
bp: base pair
C: cytosine
CNGC: cyclic nucleotide-gated channel
CPC: coding potential calculator
CRlncRNAs: cold-responsive lncRNAs
DCL1: dicer-like 1 enzyme
EST: expressed sequence tag
FDR: false discovery rate
G: guanine
GO: gene ontology
GSS: genome survey sequence
HRlncRNAs: heat-responsive lncRNAs
Hsp70: heat shock protein 70
KAT2: 3-ketoacyl-CoA thiolase 2
kcal: kitocalorie
KEGG: Kyoto Encyclopedia of Genes and Genomes
lncRNA: long non-coding RNA
Lrr: leucine-rich repeat receptor
MFE: minimal free energies
MFEI: minimal free energy index
miRNA: microRNA
mRNA: messenger RNA
NCBI: National Center for Biotechnology Information
ncRNA: non-coding RNA
ndG: normalized binding free energy
Nr: non-redundant
nt: nucleotide
ORF: open reading frame
piRNA: piwi-interacting RNA
RC12B: related cDNA 12 B
RISC: RNA-induced silencing complex
RNA: ribonucleic acid
SCL14: scarecrow-like 14
siRNA: small interfering
snoRNA: small nucleolar RNA
SPL: Squamosa promoter binding protein-like
STP13: sugar transport protein 13
UPE: maximum energy of unpairing.
